# Exosome application in treatment and diagnosis of B-cell disorders: leukemias, multiple sclerosis, and arthritis rheumatoid

**DOI:** 10.1186/s11658-022-00377-x

**Published:** 2022-09-05

**Authors:** Mohsen Karami Fath, Jalil Azami, Niloofar Jaafari, Mahsa Akbari Oryani, Nafiseh Jafari, Alireza Karim poor, Ali Azargoonjahromi, Mohsen Nabi-Afjadi, Zahra Payandeh, Hamidreza Zalpoor, Dariush Shanehbandi

**Affiliations:** 1 Department of Cellular and Molecular Biology, Faculty of Biological Sciences, Kharazmi University, Tehran, Iran; 2 Faculty of Veterinary Medicine, Urmia University, Urmia, Iran; 3 Department of Hematology and Blood Banking, Faculty of Allied Medicine, Iran University of Medical Sciences, Tehran, Iran; 4 Department of Pathology, Faculty of Medicine, Mashhad University of Medical Sciences, Mashhad, Iran; 5 Department of Microbiology, Faculty of Advanced Science and Technology, Tehran Medical Science, Islamic Azad University, Tehran, Iran; 6 School of Medicine, Tehran University of Medical Sciences, Tehran, Iran; 7 Shiraz University of Medical Sciences, Shiraz, Iran; 8 Department of Biochemistry, Faculty of Biological Science, Tarbiat Modares University, Tehran, Iran; 9 Department Medical Biochemistry and Biophysics, Division Medical Inflammation Research, Karolinska Institute, Stockholm, Sweden; 10 Shiraz Neuroscience Research Center, Shiraz University of Medical Sciences, Shiraz, Iran; 11 Network of Immunity in Infection, Malignancy & Autoimmunity (NIIMA), Universal Scientific Education & Research Network (USERN), Tehran, Iran; 12 Immunology Research center, Tabriz University of Medical Science, Tabriz, Iran

**Keywords:** Exosome, Cancer, Chronic lymphocytic leukemia, Acute myeloid leukemia, Rheumatic arthritis, Multiple sclerosis

## Abstract

Exosomes, known as a type of extracellular vesicles (EVs), are lipid particles comprising heterogeneous contents such as nucleic acids, proteins, and DNA. These bi-layered particles are naturally released into the extracellular periphery by a variety of cells such as neoplastic cells. Given that exosomes have unique properties, they can be used as vectors and carriers of biological and medicinal particles like drugs for delivering to the desired areas. The proteins and RNAs being encompassed by the circulating exosomes in B-cell malignancies are deemed as the promising sources for diagnostic and prognostic biomarkers, as well as therapeutic agents. Exosomes can also provide a “snapshot” view of the tumor and metastatic landscape at any particular time. Further, clinical research has shown that exosomes are produced by immune cells such as dendritic cells can stimulate the immune system, so these exosomes can be used in antitumor vaccines. Despite the great potential of exosomes in the fields of diagnostic and treatment, further studies are in need for these purposes to reach a convergence notion. This review highlights the applications of exosomes in multiple immune-related diseases, including chronic lymphocytic leukemia, multiple sclerosis, and arthritis rheumatoid, as well as explaining sundry aspects of exosome therapy and the function of exosomes in diagnosing diseases.

## Introduction

B-cell disorders include a highly heterogeneous class of disease. While the proportion of patients shows no symptoms and remains stable without therapy, some others indicate rapid progression of disease and need urgent treatment [1]. Emerging evidence has uncovered that, in B-cell-related malignancies such as B-cell chronic lymphocytic leukemia (B-CLL), cancerous cells require to be interacted with the tumor microenvironment (TME) to survive and proliferate [2, 3]. Actually, the growth of a tumor cell in vivo is mainly affected by the TME which comprises immune cells and the extracellular matrix constituents like cytokines and stromal components [4, 5]. Recently, “remote” communication tools of CLL cells have been discovered that they engage in novel molecular structures, namely extracellular vesicles (EVs). These particles are categorized into four categories based on their size and on the mechanism of biogenesis, all of which are secreted by all types of cells [6, 7]. The first time when EVs were discovered, they were assumed to be a kind of “rubbish bin”, to remove the unusual materials coming from exterior cells. Later, a growing body of evidence indicated that the EVs serve as functional delivery envelopes released from parental cells to other tissues. At the present, they are regarded as new actors playing a crucial role in cellular cross talks at closed and long distances facilitating cellular communications in health and disease [8, 9].

Exosomes, as one of the EVs, are responsible for the functional transfer of biologically active molecules including lipids, proteins and ribonucleic acid species (including mRNAs, miRNAs and lncRNAs) [10]. Exosomes can be secreted and uptaken by most types of cells, including immune cells. Intracellular proteins and extracellular stimuli regulate the biogenesis, secretion, and uptake of exosomes derived from immune cells. Cancer progression and metastasis can be regulated with immune cell-derived exosomes that facilitate the interaction between innate and adaptive immunity [11]. Among the immune cells, B cells are becoming increasingly clear as a potential major source of exosomes in vivo [12].It has been revealed that B cell-derived exosomes can play a key role in disease pathogenesis such as cancer via suppressing anti-tumor T cell responses [13].

Exosomes are known as double-layered lipid membrane vesicles, abundant in lipids, particularly sphingomyelin, phosphatidylserine, glycolipid GM3, and phosphatidyl ethanolamines [14]. These molecules are originated from the parental cells through the time of the exocytosis process, whereby the exosomes are able to carry their cargos throughout the bloodstream and lymph vessels to the target cells, thus affecting metabolism and phenotype of target cells [15]. As a matter of fact, the number of exosomes and also the composition of cargos may be divergent in normal and tumor situations, which is an indicator of tumor heterogeneity [16]. The content of exosomes reflects the cell activation state and can be affected by pathological processes from the origin tissue. In fact, releasing exosomes is a type of cellular adaptation mechanism, and the biogenesis compositions of exosomes and secretion thereof are influenced by the cellular microenvironment. Due to the current progress in high-throughput transcriptome assessment methods, analyzing the trace amounts of biomaterials of whole exosomes has become feasible [17].

On the exosomes secreted from antigen-presenting cells, glycosyl phosphatidylinositol-anchored proteins and also CD55 and CD59 as the complement regulators are lipid markers specific for exosomes [18, 19]. Exosomes alone are unable to perform the synthesis of functional proteins but have the ability to alter functional proteins at the first time when the cargo of RNA is transferred to a recipient cell [20]. Persuasive evidence has divulged that the regulation of exosomal mRNAs is carried out via attaching miRNA [21]. Unlike proteins or miRNA, exosomal mRNAs have not grabbed scholars’ attention to be used as disease-related biomarkers. Besides a number of technical challenges, due to selective packaging of exosome contents, exosomal mRNAs do not really represent the state of the origin cells. Furthermore, exosomal mRNAs may be exposed to degradation machinery, while there is no internal RNA control that can be taken into consideration for varied exosome yields [22]. Additionally, it has been represented that exosomes have both mitochondrial (mtDNA) and genomic (gDNA) DNA. The first DNA was envisaged to consist of one chromosome and to be capable of coding specific proteins involved in particular metabolic pathways, whereas the latter DNA is organized on the standard 46 chromosomes. Exosomal mtDNA can be found in a large number of cell types, especially glioblastoma and astrocytes cells [23]. The gDNA, denoting the total genome, is abundant in exosomes. However, there is no adequate proof of exosomal DNA, as disease biomarker. Furthermore, unlike protein and RNA molecules, DNA can be detected selectively and cell-dependently inside some exosomes. Notwithstanding having been carried out valuable surveys on exosomal DNA, mRNAs, and lipids in Rheumatoid arthritis (RA), the data provided below describe the function of the exosomal protein, miRNAs, and lncRNAs in RA[22].

**Fig. 1:**
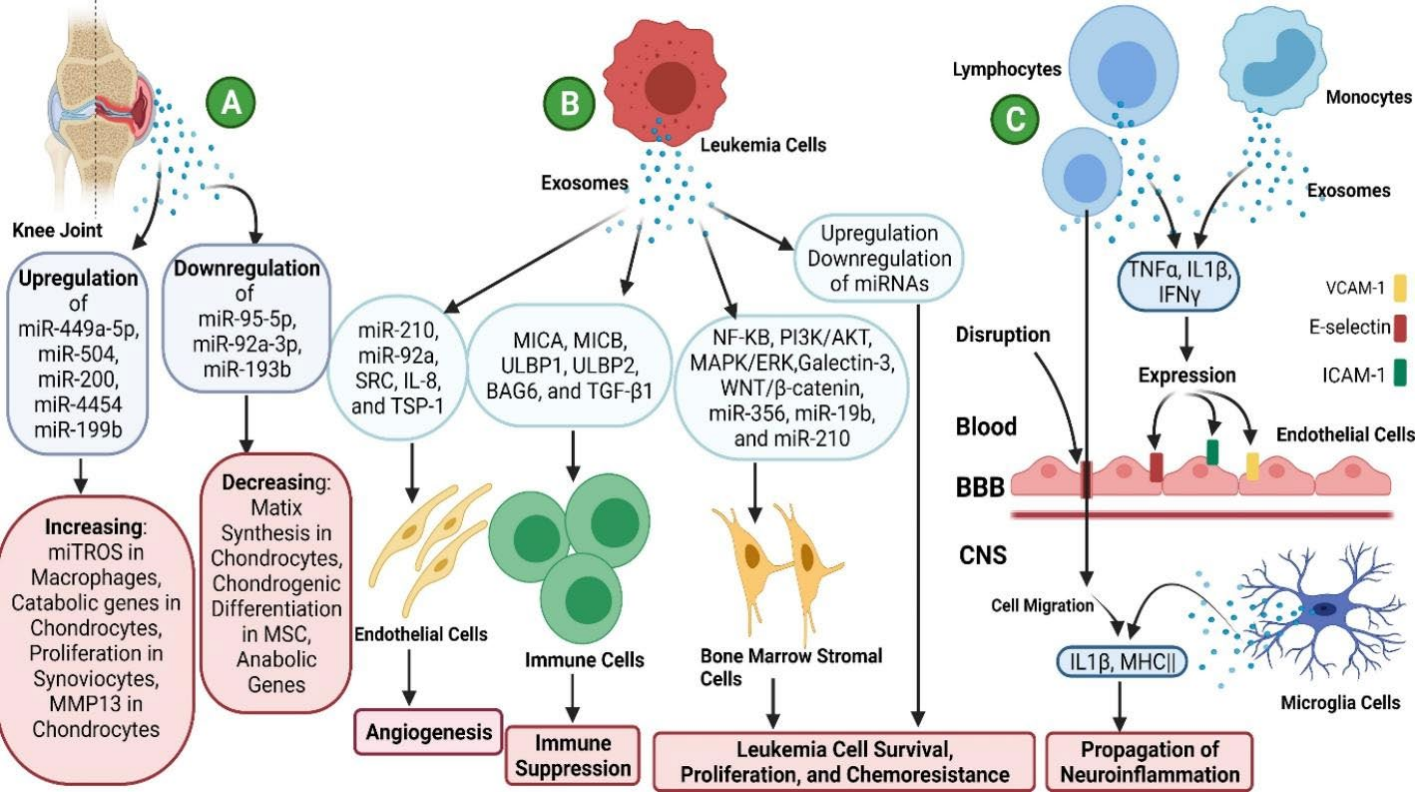
**Exosome Functions in B-cell Disorders**: A) Chronic Lymphocytic Leukemia (AML), B)Multiple Sclerosis (MS), and C) Arthritis Rheumatoid (AR)

This review highlights exosome applications in different autoimmune diseases such as multiple sclerosis (MS), RA, as well as chronic and acute lymphocytic leukemia and discusses various aspects of exosome therapy as well as the continuous attempts in this field. (Fig. 1.)

## Exosome structure and function

Exosomes are spherical 40–150 nm nano-carriers generated from various cell types, encompassing cytoplasmic fractions without organelles. These type of vesicles are surrounded by double-layered lipid membranes in orientation to the original cells. Exosomes are rich in endosomal pathway proteins via CD9, CD81, and CD82, and their secretion into the extracellular space by various cells has been observed [24]. Exosomes eliminate excess debris within the cells or establish signaling networks between cells. The biogenesis of exosomes initiates within the endosomal system. Premature endosomes are capable of maturating to late endosomes, as well as to multi-vesicular bodies (MVBs) [25]. Throughout this process, late endosomes undergo the internal budding of the membrane, and intra-luminal vesicles (ILVs) are thus created inside the large MVBs [25], which contain large amounts of cholesterol and sphingolipid rafts and clathrin. However, the endosomal sorting complex required for the transport (ESCRT) components has more ability to form ILVs, which their large numbers are secreted as exosomes, while the amount remaining of which is degraded by autophagosome complexes and cellular lysosomes [26]. The small Rab GTPases, which comprise about 70 different proteins, are capable of controlling intracellular transportation and releasing exosomes from the plasma membrane. Rab11, Rab35, and Rab27 are among Rabs that can directly control the secretion of exosomes [27].

ESCRT is composed of about 30 protein members, which are assorted into five functional sub-complexes. ESCRT-0 – the first complex – identifies ubiquitous cargos (mostly proteins and rarely RNAs) via ALIX, Vps4, and Tsg101. The second, third, and fourth complexes, i.e. ESCRT-I, ESCRT-II, and ESCRT-III, manage the budding of ILV. Vps4 – the last and fifth complex – mediates not only ultimate membrane scission but also ESCRT recycling[28]. The sorting mechanism of RNA in the exosome is a subject that has thus far remained vague and less clear-cut. However, by distributing EXO-motif, exosomal RNAs facilitate their sorting into the exosomes. The exosome loading process is affected by cellular RNA abundance. There are also different mechanisms that contribute to the adsorption of cells such as comprising macro-pinocytosis, endocytosis, phagocytosis, clathrin-dependent, and clathrin-independent endocytosis, direct binding, and ligand-receptor interplay [29]. While transmembrane integrin arrangement on exosomes is a necessary marker for tissue-specific deliverance of these vesicles [29]. Exosomes, through their exterior or loaded macromolecules, trigger a variety of signaling pathways. During cellular stress, the secretion of exosomes enhances the preservation of cellular hemostasis and limits the innate immune response. Immoderate release of exosomes inhibits the cell-cycle and results in apoptosis induction. Within cell communication networks, exosomes are central mediators in varied physiological and pathological situations, such as autoimmune and infectious diseases, neurological disorders, cancers, clouding inflammation, regenerative medicine, and tissue repair [30]. The exosome-based delivery system has exceptional merits such as stability, specificity, and safety. For delivering cargos (i.e. interfering RNA or active pharmaceuticals) in a specific approach, exosomes can reach long-distance targets. Exosomes can also act as perfect vectors being able to escape from the degradation of lysosomes and phagocytosis. After being fused with the plasma membrane, exosomes effectively deliver their freights to the intended cells. In addition, exosomes are able to remain continuously in the blood circulation without notable induction of immune responses [31, 32].

### Exosome and Cancer diagnosis

Biomarkers are defined as a “characteristic which is objectively assessed and analyzed as a marker in interventional therapy. These can be further classified as prognostic, predictive, and pharmacodynamics in the context of cancer. The normal course of cancer could be predicted by prognostic biomarkers. Response of patients to specific treatment might be assessed by using predictive biomarkers, and the efficacy of anti-tumor treatment can be measured by pharmacodynamic biomarkers [33, 34]. However, in order to be a useful clinical tool, a biomarker must have different criteria. Each cancer biomarker must represent the tumor’s characteristics, with a high degree of specificity and sensitivity, practical in usefulness, minimally invasive procedure, repeatable, and minimal cost [35]. Contents of exosomes represent the primary cells and reproduce the transcriptome and proteome of the original cell [36]. Therefore, the number of researches that study the possibility of exosomes as disease biomarkers has been increased significantly over the previous decade [37].

To summarize, exosomes are the realistic representation of the parental cell that produced them. Follow-up of circulating exosomes which carry proteins and RNAs in patients with B cell malignancy could represent a reliable source of biomarkers for prediction. Also, regular sampling could be possible because of the low invasiveness of their isolation. The progression of malignancies within patients can be tracked by assessing exosomes content, either before or after anti-cancer therapy, allowing physicians to choose the appropriate treatment option for each individual case [38]. Exosomes separated from the bloodstream of patients with head and neck cancer have protein signatures and oncogenic miRNA signatures [39]. In finding mutations in primary phase in non-small cell lung cancer (NSCLC), exosomes are more sensitive than Cell-free DNA (cfDNA) (25.7% compared to 14.2%, respectively) and more specific (96.6% and 91.7%, respectively) [40, 41]. Furthermore, a research conducted by Allenson et al., showed that a greater proportion of patients with pancreatic cancer in its early phases had more detectable kirsten rat sarcoma viral oncogene homologue (KRAS) mutations in exosomal DNA (exoDNA) than cfDNA [42].

The increase in exosome secretion by tumor cells could be due to specific mechanisms. Earlier research has demonstrated that hypoxia and stress in the TME are two reasons that could explain the increased secretions of exosome [43]. The Heparanase, p53 protein, and Rap GTPase proteins have all been found to modulate exosome production and secretion [44]. It is also been proposed that exosomes secreted by tumors in the first phase might be a possible means of early disease identification. Exosome-related protein marker was elevated in an in vivo model of pancreatic cancer before the tumor was identifiable by imaging methods [45]. By mediating biomacromolecule transfers, exosomes played a key role in mediating resistance to various therapies. Several studies in various cancers, such as breast cancer, colon cancer, and soft tissue sarcoma have shown that exosomes derived from drug-resistant cells can also make resistant in drug-sensitive cells by changing the drug-associated signaling pathways [46–49]. In short, these findings imply that tumor exosome biomarkers may have predictive and prognostic utility [50].

In cancer, exosomes are of specific interest as they are found all over the body and can be physiological or pathological bio-print. They can provide a “snapshot” view of the tumor and metastatic landscape at any particular time. The contents of exosomes, such as genetic material, are expected to be protected from degradation, enhancing the identification of clinically relevant alterations [51]. In the case of melanoma in humans, exosomes play a crucial role in modulating the antitumor immune response [52]. Zmigrodzka et al. showed by flow-cytometry that exosomes separated from plasma were higher in cancerous dogs than normal controls. This study included one melanoma-affected dog; however, it focused on exosomes released from platelets and leucocytes [53].

## Exosome application in chronic lymphocytic leukemia (B-CLL)

B-CLL is considered the most frequent type of adult hematological malignancies worldwide. People over the age of 65 are prone to be afflicted by this disease, accounting for well over 40%. The average prevalence is nearly 5 in 100,000, annually and the incidence range rises with aging. CLL is diagnosed by clinical demonstrations, changes in the number and the ratio of lymphocytes in peripheral blood smear (PBS), physical examinations, microscopic examination of bone marrow smear, and finally flow cytometric characterization of the surface marker expression of leukemic B cells [54]. CLL-related single nucleotide polymorphisms (SNPs) have been found in highly expressed proteins such as lymphoid enhancer binding factor 1 (LEF1), B-cell lymphoma 2 (BCL2), and phorbol-12-myristate-13-acetate-induced protein 1 (PMAIP1) [55]. LEF1, which is expressed in pre-B and T cells, binds to the T-cell receptor-alpha (TCRA) enhancer and confers maximal enhancer activity, and also it controls the proliferation, survival, and differentiation of hematopoietic cells [56]. In addition, BCL2 family proteins play central roles in cell death regulation and can regulate diverse cell death mechanisms including apoptosis, necrosis and autophagy. They are the key regulators of the mitochondrial pathway of apoptosis. This pathway is required for normal embryonic development and for preventing cancer [57]. PMAIP1 also plays a role in the activation of apoptosis [58]. Dysregulation of miRNAs has been shown to be associated with the onset and progression of CLL. For instance, both the onset and progression of CLL are associated with the deregulation of miR-15a and miR-16-1[59]. As the expression levels of BCL2 and the zeta-chain-associated protein kinase 70 (ZAP70) – which turns on (activates) immune system cells called T cells – gene are suppressed by miR-15a and miR-16-1, decreased expression of these miRNAs can lead to increasing BCL2 and ZAP70, which are involved in inhibiting cell apoptosis [60]. A wide variety of procedures are used in treatment of CLL. For several decades, chloramphenicol is utilized as conventional standard therapy for CLL. The use of these drugs has been shown to be related to some restrictions such as adverse effects and risk of toxicity. Over the past decade, new targeted therapeutic approaches such as bruton’s tyrosine kinase (BTK) inhibitors and Bcl2 blockers as well as innovative therapies that modulate the immune response against cancer cells [61, 62]. Non-coding RNAs have been implicated in the development and progression of CLL in numerous studies. Some of these non-coding RNAs like microRNAs (miRNAs) and tRNA-derived small RNAs (tsRNAs) have been discovered to be important biomarkers that can be used to diagnose, monitor, and predict medication resistance, assisting clinicians in selecting treatment regimens that are suited to the patient’s needs [63].

Pathogenesis of CLL is related to the tumor-supporting environment. Lymphocytic cells are highly dependent on their environment for survival, so exosomes have emerged as the main mechanism of intercellular cross-talk which provide a promising environment for stimulating CLL progression [64]. The levels of CLL-derived exosomes were considerably higher, compared to healthy patients [65]. Exosomes protein carriers carrying CD markers have been shown to act as predictive biomarkers in patients with CLL at numerous stages of the disease. Elevated levels of HLA-DR, HLA-C, HLA-B, HLA-A, CD82,CD62L, CD55, CD44, CD31, CD19, and CD5 and low levels of CD63, CD49c, and CD21 were detected by using an antibody microarray profiling of the membrane protein which content of plasma exosomes, whereas none of these changes has been shown in exosomes derived from plasma of healthy individuals [66]. In particular, CD19 and CD37 from plasma exosomes were shown highly elevated in advanced clinical stages and were associated with a relatively high cancer burden [67]. The signaling pathway of B-cell receptor (BCR) plays a vital pathogenic role in CLL [68]. Mutations in the components of the BCR signaling that give rise to the continuous activation of downstream kinases are common [68]. Exosome secretion does not show a constructive mechanism in B cells, but it is triggered by external stimuli supported by several signaling interactions [69]. Inhibitors of these stimulants can be used to prevent or inhibit the secretion of exosomes by CLL cells to prevent contact with the tumor microenvironment and to prevent tumor progression [69, 70].

## miRNAs exosomes in B-CLL

Dysregulated small non-coding RNAs are considered as major causatives of altered gene expression in cancer cells. This outstanding discovery paves the way for significant advances in the development of new and more efficient treatments for cancer. In B-CLL, the unbalanced miRNA profiles encouraged the development of anti-ROR1 antibodies and Bcl2 inhibitors [63].

Among the diverse range of molecules transported by exosomes, we will focus on miRNAs differentially expressed by CLL cells. MicroRNAs are short, highly conserved non-coding RNA molecules of about 20 nucleotides that post-transcriptionally interfere with protein expression by direct transcript degradation or translational repression. MicroRNA expression is not always fixed and depends on the source of cells and physiological or pathological situations. MiRNAs have grabbed the attention of many scientific researchers because they seem to be beneficial tools for interfering with mammalian RNA genes for in vitro studies to determine the target genes’ duty [71, 72]. The regulatory role of miRNAs on gene expression plays a key role in the initiation and progression of cancer. Tumor cells related microenvironment dictates the expression level of miRNAs to increase the expression of pro-survival genes and repress the anti-tumorigenic genes. For instance, the upregulated expression of c-Myc transacting factor in cancer is due to an increase in the expression level of a set of miRNAs that suppress their target tumor suppressor transcripts [73]. In addition, miRNAs can play as tumorigenic, so-called “oncomiRs”, while they suppress pro-apoptotic or anti-proliferative targets [74].

Several studies indicate the release of miRNAs in exosomes derived from CLL. Table 1 summarizes the most plentiful miRNAs observed in the CLL-derived exosomes and their potential roles.

**Table 1:** Exosomal miRNAs in B-CLL

mRNAs	Expression in CLL	Target	Function	Oncogenic or Suppressive	Refs.
miR-202-3p	Upregulated	Sufu gene	‘suppressing fused’ (Sufu) is targeted by gene of miR-202-3p increase, modulator of the Gli Hedgehog signaling pathway -During development, it is thought to be involved in pattern formation and cellular proliferation. - decreasing in the intracellular level of this miRNA causes an increase in the expression level of Sufu, which aids tumor cell survival and progression.	Oncogene	[69, 71]
miR-146a/miR-451	Upregulated	Kinases in Stromal cells	inducing the stromal cells activation by phosphorylation of several kinases	Oncogene	[69, 71, 75]
miR-150	Upregulated	c-Myb	playing an important role in the process of hematopoiesis, particularly in the development of lymphoid lineage	Oncogene	[69, 71]
miR-19b	Upregulated	TP53 and MKI67	TP53 downregulation and MKI67 upregulation as tumor cell proliferation, survival, and invasion mechanisms	Oncogene	[69, 76]
miR-155	Upregulated	SHIP1	In CLL, it has an oncogenic role; it promotes the BCR response by targeting SHIP1.	Oncogene	[69, 71]
miR-29	Downregulated	TCL1	regulation of TCL1, MCL1 and DNA-methyltransferases	Tumor suppressor function in aggressive CLLs (downregulated versus indolent CLL)	[69, 71]
miR-223	Downregulated	HSPs	HSP90B1	Oncogene	[69, 77, 78]
Y RNA	Upregulated		it is overexpressed in tumors and is essential for cell proliferation	Oncogene	[69, 79]

miRNAs are selected by CLL leukemic cells to be packed into the circulating exosomes to adjust the expression of their target genes in support of tumor cells’ proliferation and progression [69]. Exosomes originated from CLL cells have a high level of miR-202-3p [80, 81]. The other two miRNAs that are differentially upregulated in the CLL-derived exosomes are miR-146a and miR-451, which switch stromal cells to cancer-associated fibroblasts (CAFs) [82]. In fact, exosomes released from the CLL cells with a cargo of miR-146a and miR-451 by delivering their cargoes to stromal cells activate their target genes by phosphorylation of several kinase proteins. Furthermore, CAFs can also produce many inflammatory signals, such as cytokines and proangiogenic factors, thereby promoting tumor development and invasiveness [83]. The expression level of miRNAs (serum levels or intracellular levels) depends relatively on the phenotypic characteristics of invasive CLL. Among these miRNAs, miR-150 has a pivotal role in hematopoietic events, particularly in lymphoid lineage development and differentiation [84]. miR-150 functions are correlated with the ζ-chain-associated protein of ZAP-70 expression or the unmutated immunoglobulin heavy variable (IGHV) genes when the common molecular characteristics of poor prognosis are examined [85]. Despite the abundant upregulated expression of miR-150, either free or bound to the serum protein in circulation, CLL-derived exosomes can protect it from decomposing by RNAse, thus sustaining its pro-tumorigenic properties [86]. Interestingly, miR-155 and miR-150 expression in exosomes is positively correlated with BCR activation. miR-155 and miR-150 in serum exosomes could be valuable diagnostic biomarkers for patients with an activated BCR pathway who might take advantage of treatment with BCR-related kinase inhibitors. Moreover, serum EV-miR-155 could be a useful indicator of disease transformation from monoclonal B-cell lymphocytosis (MBL) to CLL. Ferrajoli et al. Plasma miR-155 levels of exosomes could be valuable biomarkers for the probability of transition from MBL to CLL [87].

miR-155 family is highly expressed in mature B and T cells and plays a key role in the differentiation of hematopoietic lineage, viral infections, inflammation, and cancer and immune system regulation [88]. Examination of exosomes originated from CLL shows that these vesicles have large amounts of miR-155 which can trigger and sustain the myeloid-derived suppressing cells (MDSCs) in a vitamin D-related stage [89]. The MDSCs are newly identified immature myeloid cells that are characterized by the ability to suppress immune responses and expand during cancer, infection, and inflammatory diseases [90].

A recent study has investigated circulating exosomes in CLL leukemic cells and found upregulated miR-19b as a predictor of the development of resistance to chemotherapy in CLL [91]. Yeh et al. investigated the entire collection of miRNAs that could be dysregulated in both CLL-derived exosomes and CLL cells. They discovered that miR-29 is expressed similarly in both CLL cells and normal B cells, but that CLL-obtained exosomes had elevated amounts of miR-29 than normal exosomes [68]. It seems that miR-29 plays a vital role in blood cancers by modulating T-cell leukemia/lymphoma protein 1 (TCL1), myeloid cell leukemia 1 (MCL1), and DNA-methyltransferase enzymes [92]. Some researches show that miR-223 has lowered expression in CLL-derived exosomes in comparison with healthy individuals. Moreover, miR-223 was shown to be downregulated in invasive stages of CLL and was linked with poor prognosis [93]. In vitro secreted exosomes, enriched with mt-circRNAs and upregulated mt-COX2, have been detected in a well-known CLL cell line MEC-1 [81]. The MEC1 and MEC2 are EBV-contaminated lines that were established earlier from blood-derived cells of a chronic lymphocytic leukemia (CLL) patient at different stages of progression towards prolymphocytoid transformation. In a number of ways, these lines are unique. By rearrangement of immunoglobulin genes, their common clonal origin was demonstrated. In vitro, the same indigenous strain of epstein-barr virus (EBV) caused the cells to proliferate. Their phenotypic differences are indicative of subsequent subclones emerging from the CLL population [94].

Apparently, mt-COX2 overexpression is linked to leukemogenesis and poor outcomes. This finding establishes the groundwork for the acceptance of mt-COX2 as a novel CLL prognostic biomarker [95].

## Exosome application in other Leukemia

### Exosome in chronic myeloid leukemia (CML)

Chronic myeloid leukemia (CML) is among the hematological malignancies affecting adults, with an expected prevalence of 1–2 cases per 100,000 [96, 97]. The significance of the microenvironment in leukemia development has been proven in recent years. Exosomes have been recognized as important intermediaries of communication in the midst of leukemia cells and the stromal microenvironment. Exosomes obtained from CML cells could be taken up by endothelial cells and boost endothelial cell tube formation [98]. It is worth mentioning that exosomes from K562 cell line, which are of the erythroleukemia type, can induce dasatinib-sensitive Src phosphorylation and activate downstream proteins in endothelial cells [99]. Furthermore, a study showed that premiR-92a, which originated from K562 exosomes, could decrease the expression of the target gene integrin a5 and then improve endothelial cell movement and tube formation [100]. miR-210 from CML exosomes has also been shown to interact with the target gene Ephrin-A3 and play a vital role in angiogenesis and vascular endothelial growth factor (VEGF) signaling [101].

These studies indicate how leukemia cells may transmit signals to the microenvironment and prove the curative potential of exosome-derived miRNAs in combination with presently accessible VEGF inhibitors [100, 101]. Furthermore, exosomes derived from CML can establish an autocrine loop with their parent cells via a ligand-receptor interaction. This kind of loop is mediated by exosome-associated transforming growth factor beta 1 (TGF-β1) and subsequent activation of the extracellular signal-regulated kinases (ERKs), protein kinase B (PKB), also known as AKT, and nuclear factor kappa B (NF-κB) signaling pathways, resulting in augmented CML cell expansion and survival [102].

These data show the importance of exosomes in cancer biology. In a co-culture of K562-derived exosomes with bone marrow mesenchymal stem cells (BM-MSCs) and macrophages, the expression of several genes including tumor necrosis factor alpha (TNF-α), Wnt family member 5 A (Wnt5a), interleukin-6 (IL-6), C-X-C motif chemokine 12 (CXCL12) and Dickkopf-related protein1 (DKK1) was altered. This led to increased production of nitric oxide (NO) and consequently the production of reactive oxygen species (ROS) which was reduced in a time- and dose-dependent fashion [103].

Exosomes derived from human umbilical cord mesenchymal stem cells (hUCMSC) could improve the sensitivity of K562 cells to imatinib (IM) by activating the caspase apoptotic pathway by increasing Bcl-2 associated X (Bax) expression and lowering Bcl-2 levels. Thus, a combination of IM with hUCMSC-derived exosomes may be a hopeful method to improve the efficiency of CML treatment [104]. Min QH showed that the level of miR365 was considerably higher in exosomes that come from drug-resistant CML cells in comparison with sensitive cells. In parallel, IM sensitive CML cells transfected with pre-miR365 exhibited lower chemo-sensitivity and apoptosis rate when it is compared with controls. Exosomal delivery of miR-365 may lead to drug resistance by preventing the production of pro-apoptosis proteins in non- resistant CML cells [105].

Moreover, a BCR-ABL transcript was discovered in exosomes derived from CML cell lines and CML patients sera, indicating the potential of exosomes as detection targets for BCR-ABL [106].

### Exosomes in Acute myeloid leukemia

Acute myeloid leukemia (AML) is a type of hematological malignancy that manifests rapid cellular proliferation and poor prognosis. Compared to parental cells, the presence of miRNAs was known to be greater in exosomes derived from AML cells. Nine hematopoiesis/ leukemogenesis-related factors (i.e., c-Myc, myocyte enhancer factor 2 C (MEF2C) [20], CCAAT/enhancer-binding protein alpha and -beta (CEBP-α/-β), E2F transcription factor 1 (E2F1), inhibitor of DNA binding 1 (ID1), SH2-domain-containing inositol 5′-phosphatase 1 (SHIP1), forkhead box P3 (FOXP3), and GATA-binding factor 1 (GATA1)) and five specific biomarkers (i.e., insulin-like growth factor type 1 receptor (IGF-1R), matrix metalloproteinase-9 (MMP9), C-X-C chemokine receptor type 4 (CXCR4), fms related receptor tyrosine kinase 3 (FLT3), and nucleophosmin 1 (NPM1)) associated with the prediction of AML were discovered in the relating exosomes. Kaplan-Meier analysis similarly showed that an increased miR-125b level was related to higher accumulative relapse and total death rates [107]. These data recommend possibly strong regulation of AML development by exosomes. Furthermore, higher levels of miR-223, miR-191, miR-155, miR150, miR-99b, miR-9 and let-7a (ranging from 2- to 40-fold) have been found in AML cell-derived exosomes, compared to the parental cells [108]. For instance, among mature miRNAs existed in both cellular and exosomal samples, Hornick et al. identified a set of upregulated Serum exosomal miRNAs, such as miR-150, miR-155, and miR-1246, as minimally-Invasive early biomarker of AML [109] BM-MSCs are shown to be able to uptake the exosomes released by AML cells. In an experiment, expression of CXCR4 – a target of miR150 – was significantly declined in BM-MSCs after co-culture with AML cell-derived exosomes and the movement of AML cells toward the chemokine stromal cell-derived factor-1 (SDF-1) was markedly decreased [108].

Moreover, Zhang. L et al. investigated that AML is affected by miR-425-5p loaded in exosomes derived from BM-MSCs. They indicated that the expression of miR-425-5p, participating in different cancer types, decreased in CD34 + CD38-AML cells and exosomes of AML patients, as well as AML cell lines, compared to the bone marrow healthy cases or generally healthy cases. BM-MSCs-derived exosomal miR-425-5p significantly inhibited cell viability, invasion and migration, and induced apoptosis of AML cells via upregulation of cleaved poly (ADP-ribose) polymerase (PARP) and cleaved caspase3 and downregulation of wilms tumor 1-associated protein (WTAP) [110].

AML cell-derived exosomes have also been shown to suppress the residual hematopoietic functioning preceding widespread leukemic bone marrow metastasis directly or by mediating stromal elements. Exosomes that are derived from AML cells could downregulate critical retention factors (like C-X-C motif chemokine ligand 12 (CXCL12) and stem cell factor (SCF)) in stem cells, and lead to mobilization of hematopoietic stromal and progenitor cell (HSPC) from bone marrow. On the other hand, these exosomes directly modulate HSPCs by decreasing clonogenicity and declining expression of c-Kit, CXCR4, and other hematopoietic transcription factors (i.e., c-Myc). In addition, exosomes of leukemia blasts could indirectly prohibit hematopoietic progenitor cell (HPC) activities. This suppression occurs indirectly by stromal reprogramming of niche-retention factors and re-arranges the bone marrow niche into a microenvironment which allows leukemia progression [111]. AML-derived exosomes also, can inhibit the expression of the hematopoietic factor DKK1 inducing the downregulation of hematopoietic stem cell promoters in bone marrow stromal cells and creating a favorable microenvironment for the proliferation and survival of leukemia cells. Moreover, through overexpression miR-21 and miR-29, AML exosomes promote the survival of healthy hematopoietic stem cells and induce leukemia-like functional features in them [112, 113].

Chemoresistance is a regular rampant event that happens in AML cells. Exosomes have also been found to take part in the progress of drug resistance in myeloid neoplasms [111]. AML cells are shown to release exosomes that contain VEGF/VEGFR factors that cause glycolysis in hUVECs, leading to vascular re-arrangement and chemo-resistance [114]. AML-derived plasma exosomes mediate the intercellular transmission of regulatory proteins, such as MCL-1, BCL-2- like 1 isoform 1 (BCL-XL), and BCL-2. Accordingly, these exosomes were recognized as important causes of treatment resistance, that is, exosomes have been found to have impacts on the progress of drug resistance in myeloid neoplasms [114]. Liu et al. found that PI3K/AKT/ mTOR signaling and autophagic activity were enhanced markedly in the IM-resistant CML cell line K562 (K562RIMT). Moreover, mTOR-independent beclin-1/Vps34 signaling pathway was found to have participated in exosome release from the relating cells [115]. Dasatinib can boost apoptosis by interfering with AKT/mTOR function and also stopping exosome release and preventing autophagy through decreased expression of Vps34 and beclin-1 in K562RIMT cells. In another study, the bioactivities of leukemia stem cells (LSCs) were investigated via AML-derived miR-1246. The results indicated that AML derived miR-1246 is highly-expressed and directly-targeted the human leucine rich repeats and immunoglobulin like domains 1 (LRIG1), as a negatively regulator of receptor tyrosine kinase signaling, to activate the signal transducer and activator of transcription 3 (STAT3) pathway so that, by up-regulating LRIG1, miR-1246 inhibitor/EV-encapsulated miR-1246 inhibitor can suppress the viability and colony formation abilities but promote the apoptosis and differentiation of LSCs through inactivation of STAT3 pathway [116]. Exosomal miRNAs associating in CML and AML are illustrated in Table 2.

**Table 2:** Exosomal miRNAs associating in CML and AML

miRNA	Malignancy	Alteration mode	Function/description	Refs.
Pre-miR-92a	CML	Increased	Down-regulating the integrin a5 and improving endothelial the cell movement and tube formation.	[100]
miR-210	CML	Increased	Interacting with Ephrin-A3 and playing a vital role in angiogenesis and VEGF signaling.	[101]
miR365	CML	Increased	Drug resistance by preventing the production of pro-apoptosis proteins.	[105]
miR-150, miR-155 and miR-1246	AML	Increased	Their high serum levels lead to considering them as minimally-invasive early biomarker of AML. promoting the apoptosis and differentiation of LSCs through inactivation of STAT3 pathway.	[109]
miR-425-5p	AML	Decreased	Inhibiting cell viability, invasion and migration via inducing apoptosis genes of AML cells such as cleaved PARP, cleaved caspase3 and Wilms tumor 1-associated protein (WTAP).	[110]
miR-21 and miR-29	AML	Increased	Promoting the survival of healthy hematopoietic stem cells and inducing leukemia-like functional features in them	[112, 113]

**Fig. 2:**
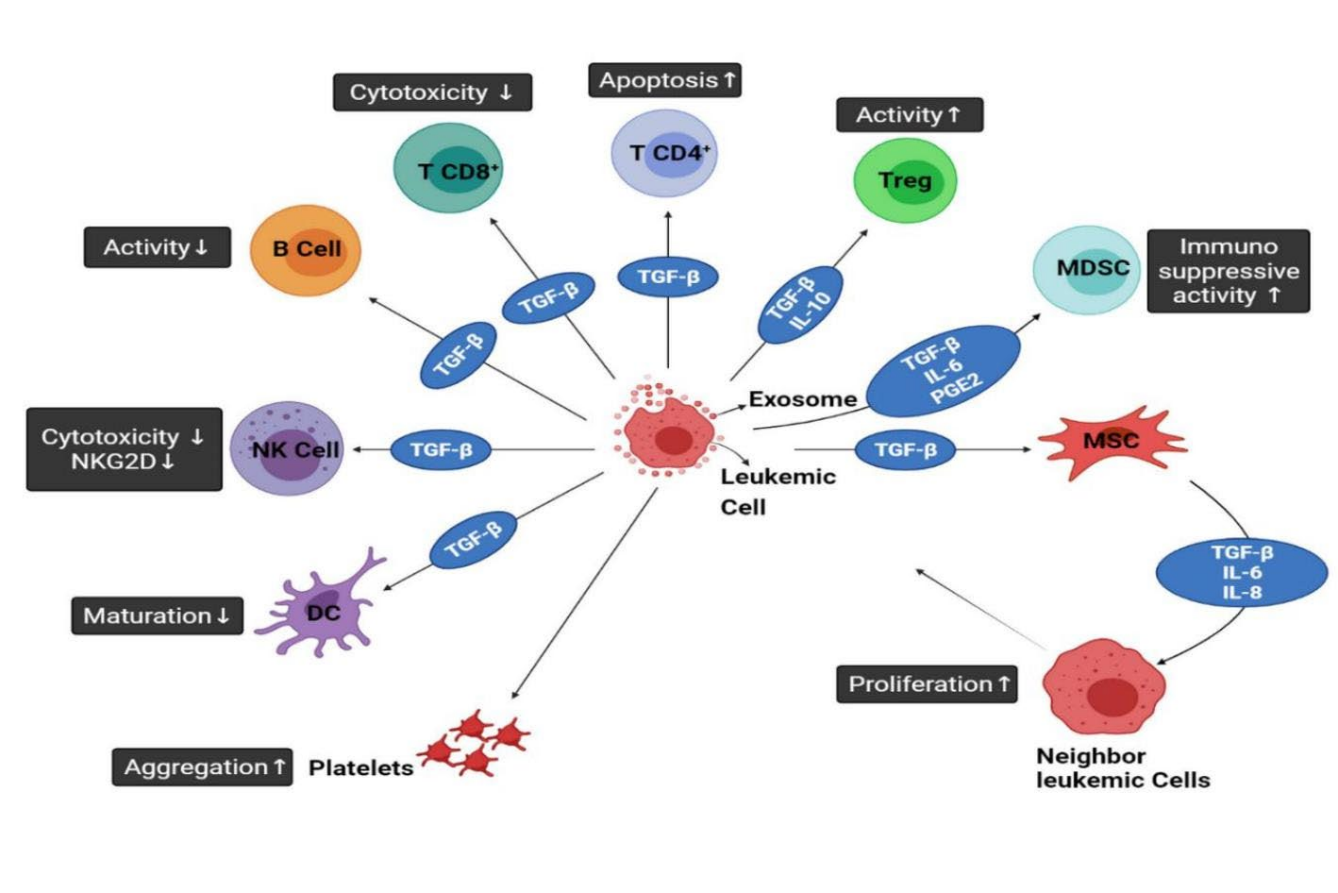
Different Effects of Exosomes Generated from Leukemic Cells on The Immune System. Furthermore, the influence of leukemia-derived exosomes and their cargoes on MSCs has been demonstrated

Szczepanski et al. [117] found that the exosomes derived from blasts in the sera of AML patients have large amounts of TGF-β1 protein and prevent the cytotoxicity of the NK cells. A potential function of exosomes in predicting response to therapy (like chemotherapy) was assayed in AML patients. It was evident that TGF-β1 levels were higher in AML exosomes. These values declined after induction chemotherapy, increased during consolidation chemotherapy, normalized during long-term and complete remission. Figure 2. shows various effects of exosomes originated from leukemic cells on immune system. As demonstrated in Fig. 2, one of the reasons for progression of leukemia is caused by the fusion of late endosomes with the cytoplasmic membrane and, subsequently, the production of exosomes by a leukemic cell. These exosomes are contained molecules such as TGF-β that promote leukemia. Exosomal TGF-β decreases cytotoxicity and activity and increases apoptosis in CD4^+^ and CD8^+^T cells. TGF-β has also similar effects on the activity of B cells. TGF-β shows its effect on natural killer cells (NK cells) by reducing the number of surface receptors NKG2D (C-type lectin-like receptors) and cytotoxicity. The presence of this receptor on NK cells and the presence of its ligand on cancer cells cause cancer cells to lysis. Therefore, lowering of NKG2D plays a critical role in escaping the immune system. TGF-β plays an important role in escaping of leukemic cells from the immune system by preventing of dendritic cells (DCs) cells from maturing. TGF-β affects regulatory T cells (Treg cells) by increasing their activity, and also it boosts the activity of MDSCs along with prostaglandin E2 (PGE2) and IL-6.

## Correlation between Exosome and multiple sclerosis (MS)

Multiple sclerosis (MS) is an autoimmune disease, defined by chronic inflammation, demyelination, leading to neurological impairment [118, 119] The most common cause of neurologic dysfunction in young adults is MS [120]. Inflammation, demyelination and neuro-axonal injury are renowned features of MS that affect the central nervous system and cause long-term persistent disability [120]. Treatment choices for MS are limited and include common immunosuppressives, immunomodulatory agents, or compounds that inhibit lymphocyte infiltration into the CNS [121].

The raid of auto-reactive CD4^+^ T cells (especially Th17and Th1 cells) into the CNS, is a main immune-pathologic characteristic of multiple sclerosis, leading to neuro-axonal degeneration and disability. According to the literature, microRNAs also play crucial roles in the pathology of MS [74, 122, 123]. Since exosomes can cross the blood-brain barrier, it is likely that some circulating exosomes originated from affected CNS cells or the related inflammatory microenvironment be engaged in MS patients [124, 125]. Cells’ exosomes are major carriers of miRNAs into the circulation protecting them from degradation. Accordingly, exosomal miRNAs seem to be more stable than free miRNAs and leukocyte-derived exosomal miRNAs could provide useful information about MS pathogenesis [126–128]. Ebrahimkhani et al. assumed that physiological alterations linked to the initiation and progression of MS are reflected in a variety of exosomal miRNAs in serum. They revealed that serum exosomal miRNA profiles can accurately identify MS patients from healthy controls, as well as relapsing-remitting multiple sclerosis (RRMS) from progressive disease by next-generation sequencing (NGS) and integrative bioinformatics [129].

Additionally, the development of Th1 and Th17 inflammatory cells has been shown to be induced by miR-155 and mir-326. Du et al. indicated that mice lacking MicroRNA-155 are resistant to experimental autoimmune encephalomyelitis (EAE) [130]. Some miRNAs like miR-146a and miR-29, on the other hand, have been linked to the prevention of autoimmune disorders. MicroRNA-146a inhibits Th1 cell activity by activating regulatory T cells [122, 123]. Moreover, the production of gamma interferon (IFN-γ) is suppressed by miR-29 and miR-29 deficient cells produce significantly more IFN-γ compared to normal cells [131]. In addition, a dysregulated miRNA profile is evident in leukocytes of MS patients [132, 133]. Keller et al. demonstrated that among patients with RRMS, 165 miRNAs were substantially up- or down-regulated in comparison with healthy controls [134]. Similarly, miR-106b and miR-25, which can potentially regulate TGF-β signaling pathway, have lowered expression in regulatory T cells of MS patients [135]. Thus, the pathology of MS may be affected by the alteration of miRNA expression. A recent study by Azimi et al., found an altered abundance of miR-326 in exosomes derived from conventional T cells from MS patients [136]. It has been previously shown that miR-326 has a prominent role in the immunopathogenesis of MS via Th17 cells maturation and differentiation [130]. Consequently, exosomal miR-326 may be a clinical goal in the course of MS. Additionally, deregulation of this miRNA may be used as a diagnostic and prognostic biomarker [137].

Dendritic cells (DCs), the important modulators of adaptive immune reactions, have recently been known as scalable sources of exosomes in vitro in MS. Depending on the external factors and cell conditions, the composition of DC exosomes differs from one another. For instance, the profiles of exosomal miRNAs differ noticeably between mature and immature lipopolysaccharide-stimulated DCs [138].

Pusic et al. have found that phasic stimulation with low-level IFNγ could significantly improve myelination in cultured brain slices or following nasal administration to animals. Then, they used low-level IFNγ for primary DC cultures stimulation and assessed the miRNA affecting the function of exosomes [139]. Previous experiments have demonstrated that peripheral exosomes can possibly affect brain myelination through the delivery of miR-219 [139]. It was deduced that exosomes released by IFN-stimulated DCs (IFNγ-DC-Exos) can improve the myelination process and oxidative tolerance. Additionally, administration of IFNγ-DC-Exos in slice culture increased recovery from MS-like demyelination. It was endorsed that nasally administered IFNγ-DC-Exos can improve brain myelination in vivo. In vitro tracking studies indicated that oligodendrocytes and microglia (to a smaller degree) preferentially absorb IFN-DC-Exos. This is an elementary step in understanding the mechanisms of exosome-mediated myelin development, and the introduction of exosomes as therapeutic agents for re-myelination [140].

The understanding of miRNA’s role in MS has advanced remarkably over the last decade. In a pioneering study, miR-155 was recognized as the main regulator of inflammatory reactions. In mice, targeted deletion of this gene led to a decrease in cellular differentiation of Th1 and Th17 in both the CNS and peripheral lymphoid organs. Also, miR-155 deletion and pharmacological targeting both resulted in a delay in the course and severity of EAE [141]. In another investigation, miR-155-3p was discovered to cause the development of autoimmune demyelination by regulating heat shock protein (HSP) [142]. MiR-301a also was found to have a central role in the regulation of myelin-reactive T-helper type 17 cells, supporting the role of miRNAs as therapeutic agents for regulating autoimmune demyelination [143]. Beside their function in regulating the responses of effector inflammatory T cells, they also play role in modulating the Treg cells population which in normal counterbalances the former. Surprisingly, Treg formation and function have also been affected by miR-155, to such an extent that miR-155 is regulated by Foxp3 which is a characteristic transcription factor for this cell type [144]. The intricacy of the immune system’s regulatory networks is highlighted by the opposite functions of the same miRNA in different cells. While the regulating role of miRNA in pro-inflammatory responses during EAE was highlighted in the preceding cases, further research also revealed their main role in regulating myelination itself. For instance, myelin thickness has been increased in mice by overexpressing miR-23a, and also studies have shown that the synthesis of myelin and of oligodendrocyte differentiation are both increased by miR-23a in vivo [145].

Despite having been discovered many drug delivery systems such as chemical small-molecule drugs, protein drugs, or nucleotide drugs, exosomes have been demonstrated to have a lot of use in modern medicine [146]. It has been shown that exosomes are able to cross the blood brain barrier (BBB) and responsible for spreading brain antigens to the periphery. Microglia-derived exosomes were injected into the central nervous system (CNS) of EAE animals, which increased inflammation and exacerbated illness [147].

Mesenchymal stem cells (MSCs) stimulated by IFNγ produced exosomes have been demonstrated to have a noticeable effect on treating EAE. These findings showed that exosomes derived from MSCs can be employed as cell-free treatments for autoimmune and CNS illnesses [148]. Application of exosomes derived from overexpression of TGF-1 dendritic cells for the treatment of EAE leads to enhanced Treg production and suppressed Th1 and Th17 differentiation, resulting in a weakened intensity of EAE [149]. Casella G. et al. designed a mouse microglial cell line secreting high quantities of engineered exosomes carrying the anti-inflammatory cytokine IL-4. Following the administration of the mentioned exosomes, the clinical score of EAE was dramatically declined [150]. Also nasal administration of curcumin-loaded exosomes in mice with lipopolysaccharide (LPS)-induced encephalitis led to a reduction of neuroinflammation by targeting microglia [151]. Moreover, exosome-encapsulated curcumin exhibited improved anti-inflammatory effects in LPS-induced inflammatory demyelinating models [152].

Pusic et al. pointed out that exosomes generated by bone marrow-derived dendritic cells could be used as a therapeutic medium for oligodendrocyte maturation [125]. In vivo studies showed that the maturation of oligodendrocytes and recovery of myelin damage could be promoted by using these exosomes. Accordingly, exosomes may not only aggravate inflammation during EAE but also promote myelin regeneration [146].

New data on the use of miRNAs and other molecular cargos carried by circulating exosomes as biomarkers for a variety of diseases has attracted a great deal of interest in the significance of these microvesicles as a possible indicators of MS state and disease monitoring [153] (Table 3.). Interestingly, exosomes in the blood reveal the presence of CNS markers originated from the brain [154]. The analysis of these exosomes could provide an exclusive tool for understanding the status of the CNS and eliminate the need for biopsy [155]. Furthermore, exosomes could be a source of the BBB-crossing CNS antigens [154] that could provoke more investigations into exosome analysis for understanding CNS conditions and MS [156].

**Table 3:** Exosomal mRNA as biomarkers in MS

mRNA	Source of exosomes	Function	Refs.
miR-326	Conventional T cells	differentiation and maturation of Th17	[136]
let-7i	Plasma	inhibit the initiation of regulatory T cells	[157]
miR-15b-5p	Serum	Targets FGF-2 implicated in Demyelination and remyelination	[158]
miR-451a	Serum	Regulator of oxidative stress	[158]
miR-30b-5p	Serum	Neuro-axonal injury	[158]
miR-342-3p	Serum	Neuro-axonal injury	[158]
miR-122-5p,miR-196b-5p,miR-301a-3p,miR-532-5b	Serum	Targets STAT3 and AHR, differentiation and regulation of Th17	[159]

## Exosome and rheumatic arthritis

Rheumatoid arthritis (RA) is an autoimmune inflammatory disease that involves 0.5-1% of the world’s adults [160]. Women are three times more vulnerable than men, and the highest frequency of this autoimmune disease is between the ages of 40–50. Rheumatoid arthritis usually causes joint damage as well as disability. Early diagnosis and treatment may prevent joint destruction and also achieve good results in the long term. Because most irreversible joint damage occurs in the first two years of illness, optimal disease management is essential in the first three to six months [161–164]. Therefore, a reliable biomarker is needed for the timely diagnosis of the disease and effective disease management. Some features of RA are infiltration of immune cells in the joints, joint pain, and bone erosions. Different auto-antibodies have been identified based on their antigen type in RA disease that the two most common types of these auto-antibodies are anti-citrullinated protein antibodies (ACPA) and rheumatoid factor (RF). Like other rheumatoid diseases, many factors are engaged in the pathogenesis of RA, including environment, genetic background, and serological elements. It has been found that the effect of genetics is about 50 to 60%, so genetic factors play an important role in the development of RA disease. Most of the genes that cause RA, which account for about 30 to 50% of the genetic factors involved in causing RA, belong to human leukocyte antigen (HLA) genes [164–167]. Multiple risk alleles associated with RA which are located in the HLA-DRB1 gene cause the production of a conserved amino acid sequence. Recent advances in genome wide association studies (GWAS) have enhanced our understanding of the influence of genetic factors on the development of RA, and also revealed that RA is a polygenetic disease. These studies have linked more than 100 genetic loci that enhance the risk of developing RA [165, 168–171]. One of these important genes that plays an important role, especially in Japanese populations, is the peptidyl arginine deiminase 4 (PADI4) gene. Also, an R620W mutation in PTPN22 is another missense risk variant in RA disease. PTPN22 encodes a protein tyrosine phosphatase in hematopoietic cells. In T and B cells, this enzyme works as a negative modulator of the antigen receptor signaling pathways. The R620W risk allele is a gain-of-function mutation that attenuates TCR and BCR signaling pathways in cells that carry this risk allele. The HLA locus has been linked to higher serum levels of antibodies against citrullinated protein and has been linked to seropositive RA [165, 168–171].

Moreover, there are some biological treatments for RA that target inflammatory factors like TNF-α. These antibodies and proteins are mostly sedative and cannot reverse the symptoms of RA. Recently, gene therapy has yielded encouraging preliminary outcomes; nonetheless, its different aspects are still under investigation [172]. Numerous studies have shown that vesicles can be good carriers of drugs due to their unique properties. Exosomes can deliver their drug cargoes to targets because of their ability to cross different biological membranes like synovial membranes [172]. In the blood stream, exosomal packed curcumin has a longer half-life than free curcumin. Therefore, encapsulation of the drugs in the exosomes increases the half-life of the drugs [173]. Thus, in inflammatory diseases and RA, exosomes seem to be promising transfer tools for a variety of drugs. For example, in collagen-induced arthritis (CIA) mice in which miR-146a/miR-155 was downregulated, the miR-146a/miR-155 transduced MSC-derived exosomes significantly altered the Treg cells levels of CIA mice cell and recovered their appropriate responses [174] A similar study was done on therapeutic effect of MSC–derived miR-150-5p exosomes (Exo-150) on collagen-induced arthritis mice. The results revealed that injection of Exo-150 reduced hind paw thickness and the clinical arthritic scores in modeled mice. Moreover, Exo-150 decreased migration and invasion in RA fibroblast like synoviocytes (FLS) and downregulated tube formation in HUVECs by targeting MMP14 and VEGF [175].

Exosomes can deliver anti-TNF-α and other anti-inflammatory drugs to their destination without being degraded by different enzymes. In this line, some features of exosomes, such as immunosuppressive and anti-inflammatory properties, have been identified in different diseases, such as CIA and inflammatory bowel disease (IBD) [176, 177].

**Fig. 3:**
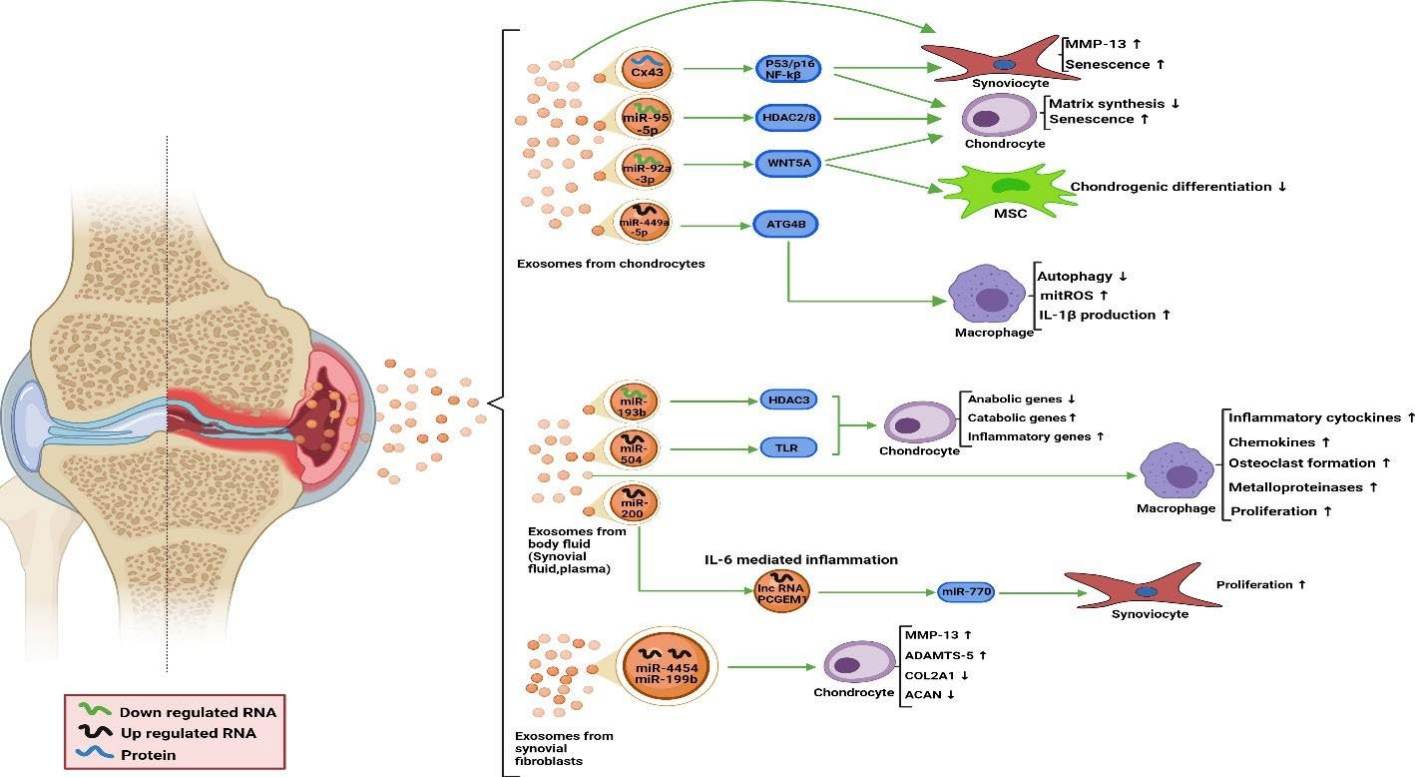
Exosomes involved in pathology of RA

Numerous studies have shown that exosomes play effective roles in cell-cell communication (Fig. 3.).

Furthermore, exosomes are involved in other activities such as the transfer of genetic information and signaling compounds, antigen presentation, and immune regulation. Exosomes transport receptors and ligands, as well as peptide-MHC complexes and antigenic material, so they play an effective role in regulating immune responses. It has shown that exosomes found in fluid are released by cells that have penetrated knee fluid. Different cells that infiltrated into the synovial joint release exosomes into the synovial fluid to injury the joint and sever the RA adverse symptoms. These exosomes can be contained different miRNAs, such as hsa-miR-151a-3p, hsa-miR-199a-5p, hsa-miR-370-3p, hsa-miR-589-5p, and hsa-miR-769-5p, to be connected with the common pathogenesis of RA [178].

Approximately, in the synovial fluid of people with inflammatory arthritis such as RA, the most common exosomes found are originated from platelets [179]. In addition, the presence of exosomes derived from neutrophils, monocytes, and granulocytes has been described. According to the literature, B and T cells could release exosomes in the synovial fluid, inasmuch as exosomes extricated from T cell are more abundant in the RA synovial fluid and have a significant correlation with rheumatoid factor serum levels [179].

human T lymphocytes also, can release exosomes containing numerous amounts of miR-204-5p which can be transferred into synovial fibroblasts and inhibit cell proliferation. Exosomal miR-204-5p expression is inversely correlated with disease parameters of RA patients, such as rheumatoid factor, erythrocyte sedimentation rate, and C-reactive protein [180].

In general, exosomes that have been derived from synoviocytes, in inflammatory diseases such as RA, promote adjacent cells to release inflammatory agents and cause cartilage damage. It has been revealed that dendritic cells treated with IL-10 can release exosomes which reduce the severity of RA. Exosomes derived from IL-1-stimulated fibroblast-like synoviocytes are shown to induce the osteoarthritic alterations in chondrocytes [181]. In RA models, exosomes have immunological activities such as motivating apoptosis, antigen-presenting to T cells, and extracellular damages [182]. This evidence confirms the use of synovial-derived exosomes as a RA biomarker to anticipate disease stages and to develop novel and more effective treatments. The pathogenesis of RA may be due to exosomes which establish cell-to-cell communication and participation thereof in many functions such as inflammation, delivery of miRNAs, destruction of extracellular matrix, antigen presentation, and formation of immune complexes [183]. It can be concluded that exosomes make a connection between synoviocytes, immune cells, chondrocytes, and endothelial cells, so they affect different processes of RA. Taken together, these findings identify the role of exosomes in the pathogenesis of RA and may be utilized to develop new therapeutic solutions [181, 184].

Exosomes could change the joint structure by various functions including antigen presentation, angiogenesis, regulation of inflammation, alteration in cell-to-cell communication, and extracellular matrix degradation [185]. Exosomes derived from RA FLS may induce CD4^+^ T cells by interfering with activation-induced cell death, fortified IL-2 and IFNγ release from the mentioned cells, increasing the activity of Akt and NF-κB, and preventing caspase 8 and caspase 3 degradation [181, 186]. RA FLS secrets exosomes that destroy cartilage and bone by autocrine activation of matrix metalloproteinase 1 (MMP-1) or direct activity of metalloproteinase. On the other hand, TNF activation of RA FLS increases the production of exosomal miR-221-3p, which in turn inhibits bone formation [187, 188]. In addition to exosome secretion, FLS can target exosomes secreted by other immune cells like monocytes and activated T cells. Under this condition, FLS might increase the releasing of MMPs, also chemokines and cytokines, like monocyte chemotactic protein 1 (MCP-1), MCP-2, IL-8, and IL-6. Moreover, transcription of CXCL6, CXCL5, CXCL3, CXCL2, and CXCL1 chemokine genes may be increased [184]. Exosomes derived from synovial fluid might act as receptors for activators of NF-κB and receptor activator of nuclear factor kappa-Β ligand (RANKL), which can activate the functioning of bone osteoclasts and resorption [189]. Exosomes derived from the plasma of RA patients with severe disease activity enhanced proinflammatory cytokines such as TNF, IL-17, and IL-1 generated by autologous peripheral blood mononuclear [181]. Furthermore, the increased amounts of C1q suggested that circulatory exosomes in RA expand the inflammation by transporting complement components. In the connection between inhabitant synoviocytes like FLS and immune cells in a joint with inflammation, exosomes are secreted into the synovial tissue and fluid. The increased amount of released proinflammatory cytokines will activate proliferation, immune cell infiltration, and angiogenesis. Thus, exosomes provide positive feedback throughout the inflammatory stage. Joint inflammation may enhance the infiltration of neutrophil-derived exosomes, which may reduce the additional degradation of cartilage in RA [190].

It had been shown that both adaptive and innate immunological responses are regulated by exosomes generated by DCs. Genetic alteration and reaction modulated by cytokine/cytokines inhibitors might lead to creating immunosuppressive and tolerogenic immature DCs. Efficacy of exosomes derived from genetically engineered immature DCs that overexpress Th2 cytokine (such as IL-4) has been found to be effective for CIA therapy model illness [191, 192]. Humoral immunity responses could be decreased effectively by these exosomes and when activated by an antigen and immune cells in the spleen become activated and result in a Th-2 dominant response [191].

DCs which express FasL are efficient at preventing collagen reactive T cell activation and decreasing CIA progression in mice. In the murine delayed-type hypersensitivity (DTH) model, exosomes released by these DCs are found to have anti-inflammatory properties. This kind of DTH response suppression is specific to the antigen and depends on class II MHC. Moreover, the efficacy of DC/FasL exosomes in the treatment of CIA murine models has been shown by systemic injection. During animal DTH and CIA treatment, it has been demonstrated that exosomes originated from immunosuppressive DCs have an equivalent or stronger effect compared to exosomes of the primary DCs, so they could potentially be utilized for the treatment of RA. In addition to the effectiveness of DCs ex vivo, exosomes produced by DCs are more stable throughout the isolation procedures and seem to be safer for in vivo delivery than autologous cells. Exosomes have a longer lifetime and due to their unique biologic properties, they are more detectable after injection in comparison with other cell types [177, 191]. Exosomes generated by immature DCs were studied for their effectiveness as a cell-free treatment for CIA in mice. It was discovered that immature DCs treated by IL-10 produced exosomes that inhibited inflammatory and autoimmune reactions through suppressing the pro-inflammatory cytokines IL-1 and TNF-α and decreased the levels of Hsp70. It was reported that DTH responses in contralateral joints were reduced by these exosomes via modulation of T cell responses [176]. In addition, exosomes from DCs treated with IL-10 could prevent the incursion of murine CIA and also reduce the severity of existing arthritis. Other research showed that inflammation and autoimmunity were suppressed by exosomes produced by DCs. This suppression is done through the regulation of both endogenous T cells and APCs activity, MHC class II-dependent, and an antigen-specific pathway. Exosomes generated from DCs had a similar or even stronger immunosuppressive impact in comparison with their original secreting cells [176, 193]. It has been revealed that the indoleamine-pyrrole 2, 3-dioxygenase (IDO) enzyme which degrades tryptophan, has an immunosuppressive capability in a variety of conditions, such as RA. Although the main mechanism of this effect has not been completely discovered, it has been proposed to be via preventing the CD8^+^ effector T cell response and induction of Treg cells, by either depriving them of essential tryptophan. Importantly, immunosuppressive exosomes with higher levels of stability and proper bioactivity for delivery could be released by DCs which express IDO [194]. On the other hand, there are other findings that demonstrated that exosomes may have a pro-inflammatory ability in RA, unlike the anti-inflammatory exosomes mentioned above. For instance, in a study by Lim. M et al. it was indicated that Has-miR-1915-3p and has-miR-6511b-5p were significantly higher in the serum exosomes of RA disease so that the level of serum C-reactive protein (CRP) was negatively correlated with them [195].

## Conclusion

The emergence of exosomes and their different activities has undoubtedly been one of the most important discoveries in the fields of medical and biology sciences in recent years. Given that exosomes have unique biological characteristics, they have been noticed in the treatment and diagnosis of various disorders and diseases, in particular B-cell disorders such as Leukemia, Multiple Sclerosis, and Arthritis Rheumatoid. Some characteristics of exosomes include: unique physicochemical properties and structure, low toxicity and immunogenicity, efficient cellular entry, inherent targeting, innate ability to cross biological barriers, and high stability in blood circulation. Exosomes play important roles in the maintaining of normal physiological conditions as well as disease status. Therefore, the type of exosomes released in health conditions are different from ones secreted in disease states; this feature can be utilized as an advanced strategy in precise and personalized diagnosis and treatment of varied disorders such as B-cell disorders. Exosomes, owing to their unique properties, can be used as vectors and carriers of biological and medicinal particles like drugs for delivering to the desired areas. Clinical research has shown that extracellular vesicles produced by immune cells (such as dendritic cells) can stimulate the immune system, so these exosomes can be used in antitumor vaccines. Exosomes are closely related to the survival, proliferation, metastasis, and recurrence of tumors, and they play an important role in the diagnosis and pathological identification of tumors and provide new avenues for immunotherapy. Despite the great potential of exosomes in the fields of diagnostic and treatment, further researches are in need for these purposes, so complementary cooperation between physicians and biologists can shed further light on the basic functions of extracellular vesicles and their different applications.

## Data Availability

The datasets used and/or analyzed during the current study are available from the corresponding author on reasonable request.

## Abbreviations

**B-CLL**: B-cell Chronic Lymphocytic Leukemia.
**TME**: Tumor Microenvironment.
**EVs**: Extracellular Vesicles.
**mtDNA**: Mitochondrial DNA.
**gDNA**: Genomic DNA.
**RA**: Rheumatoid Arthritis.
**MS**: Multiple Sclerosis.
**AML**: Chronic Lymphocytic Leukemia.
**MVBs**: Multi-vesicular Bodies.
**ILVs**: Intra-luminal Vesicles.
**ESCRT**: Endosomal Sorting Complex Required for the Transport.
**NSCLC**: Non-small Cell Lung Cancer.
**cfDNA**: Cell-free DNA.
**KRAS**: Kirsten Rat Sarcoma Viral Oncogene Homologue.
**exoDNA**: Exosomal DNA.
**PBS**: Peripheral Blood Smear.
**SNPs**: Single Nucleotide Polymorphisms.
**LEF1**: Lymphoid Enhancer Binding Factor 1.
**BCL2**: B-cell Lymphoma 2.
**PMAIP1**: Phorbol-12-myristate-13-acetate-induced Protein 1.
**TCRA**: T-cell Receptor-alpha.
**ZAP70**: Zeta-chain-associated Protein Kinase 70.
**BTK**: Bruton’s Tyrosine Kinase.
**miRNAs**: microRNAs.
**tsRNAs**: tRNA-derived Small RNAs.
**BCR**: B-cell Receptor.
**CAFs**: Cancer-associated Fibroblasts.
**IGHV**: Immunoglobulin Heavy Variable.
**MBL**: B-cell Lymphocytosis.
**MDSCs**: Myeloid-derived Suppressing Cells.
**TCL1**: T-Cell Leukemia/Lymphoma 1.
**MCL1**: Myeloid Cell Leukemia 1.
**CLL**: Chronic Lymphocytic Leukemia.
**EBV**: Epstein-Barr Virus.
**CML**: Chronic Myeloid Leukemia.
**VEGF**: Vascular Endothelial Growth Factor.
**TGF-β1**: Transforming Growth Factor Beta 1.
**ERKs**: Extracellular Signal-regulated Kinases.
**PKB**: Protein Kinase B.
**NF-κB**: Nuclear Factor Kappa B.
**BM-MSCs**: Bone Marrow Mesenchymal Stem Cells.
**TNF-α**: Tumor Necrosis Factor Alpha.
**Wnt5a**: Wnt Family Member 5 A.
**IL-6**: Interleukin-6.
**CXCL12**: C-X-C Motif Chemokine 12.
**DKK1**: Dickkopf-related Protein1.
**NO**: Nitric Oxide.
**hUCMSC**: Human Umbilical Cord Mesenchymal Stem Cells.
**IM**: Imatinib.
**Bax**: Bcl-2 Associated X.
**AML**: Acute Myeloid Leukemia.
**MEF2C**: Myocyte Enhancer Factor 2 C.
**CEBP-α/-β**: CCAAT/Enhancer-binding Protein Alpha and -Beta.
**E2F1**: E2F Transcription Factor 1.
**ID1**: Inhibitor of DNA Binding 1.
**SHIP1**: SH2-domain-Containing Inositol 5′-phosphatase 1.
**FOXP3**: Forkhead Box P3.
**GATA1**: GATA-binding Factor 1.
**IGF-1R**: Insulin-like Growth Factor Type 1 Receptor.
**MMP9**: Matrix Metalloproteinase-9.
**CXCR4**: C-X-C Chemokine Receptor Type 4.
**FLT3**: Fms Related Receptor Tyrosine Kinase 3.
**NPM1**: Nucleophosmin 1.
**SDF-1**: Stromal Cell-derived Factor-1.
**PARP**: Poly (ADP-ribose) Polymerase.
**WTAP**: Wilms Tumor 1-associated Protein.
**SCF**: Stem Cell Factor.
**HSPC**: Hematopoietic Stromal and Progenitor Cell.
**HPC**: Hematopoietic Progenitor Cell.
**LSCs**: Leukemia Stem Cells.
**LRIG1**: Leucine Rich Repeats and Immunoglobulin Like Domains 1.
**STAT3**: Signal Transducer and Activator of Transcription 3.
**NK cells**: Natural Killer Cells.
**Treg cells**: Regulatory T Cells.
**PGE2**: Prostaglandin E2.
**MS**: Multiple Sclerosis.
**RRMS**: Relapsing-remitting Multiple Sclerosis.
**NGS**: Next Generation Sequencing.
**EAE**: Experimental Autoimmune Encephalomyelitis.
**IFN-γ**: Gamma Interferon.
**DCs**: Dendritic Cells.
**IFNγ-DC-Exos**: Exosomes Released by IFN-stimulated DCs.
**HSP**: Heat Shock Protein.
**BBB**: Blood Brain Barrier.
**CNS**: Central Nervous System.
**MSCs**: Mesenchymal Stem Cells.
**LPS**: Lipopolysaccharide.
**RA**: Rheumatoid Arthritis.
**ACPA**: Anti-citrullinated Protein Antibodies.
**RF**: Rheumatoid Factor.
**HLA**: Human Leukocyte Antigen.
**GWAS**: Genome Wide Association Studies.
**PADI4**: Peptidyl Arginine Deiminase 4.
**FLS**: Fibroblast Like Synoviocytes.
**IBD**: Inflammatory Bowel Disease.
**MMP-1**: Matrix Metalloproteinase 1.
**MCP-1**: Monocyte Chemotactic Protein 1.
**RANKL**: Receptor Activator of Nuclear Factor Kappa-Β Ligand.
**DTH**: Delayed-type Hypersensitivity.
**IDO**: Indoleamine-pyrrole 2, 3-Dioxygenase.
**CRP**: C-reactive Protein.
